# Prognostic significance of ^18^F-FDG PET/CT parameters in soft tissue sarcoma: a systematic review and meta-analysis

**DOI:** 10.1186/s40644-025-00912-x

**Published:** 2025-07-29

**Authors:** Shaoli Li, Rui Bai, Hui Wang, Qunan Sun, Guannan Wang, Sujing Jiang, Ying Dong

**Affiliations:** 1 Department of Medical Oncology, the Second Affiliated Hospital, Zhejiang University School of Medicine, Hangzhou, 310009 China; 2 Cancer Institute, Key Laboratory of Cancer Prevention and Intervention, the Second Affiliated Hospital, Ministry of Education, Zhejiang University School of Medicine, Hangzhou, 310009 China; 3 Cancer Center, Zhejiang University, Hangzhou, 310009 China; 4 Department of Pathology, Institute of Basic Medicine and Cancer (IBMC), The Cancer Hospital of the University of Chinese Academy of Sciences (Zhejiang Cancer Hospital), Chinese Academy of Sciences, Hangzhou, 310022 China; 5 Department of Geriatrics, Xiaoshan Geriatric Hospital, Hangzhou, 311200 China; 6 Department of Medical Oncology, Cancer Hospital & Shenzhen Hospital, Chinese Academy of Medical Sciences and Peking Union Medical College, Shenzhen, 518116 China; 7 Department of Gastroenterology, The Second Affiliated Hospital, Yuying Children’s Hospital of Wenzhou Medical University, Wenzhou, 325027 China

**Keywords:** ^18^F-Fluorodeoxyglucose positron emission tomography/computed tomography, Prognosis, Prediction, Soft tissue sarcoma, Meta-analysis

## Abstract

**Background:**

The role of ^18^F-fluorodeoxyglucose positron emission tomography/computed tomography (^18^F-FDG PET/CT) parameters to predict prognosis for patients with soft tissue sarcoma (STS) remains controversial.

**Objectives:**

This meta-analysis aimed to systematically evaluate the prognostic significance of ^18^F-FDG PET/CT parameters in STS.

**Design:**

This study is a systematic review and meta-analysis.

**Data sources and methods:**

A literature search was conducted in PubMed, Embase, and the Cochrane Library for relevant studies up to January 1st, 2024. Studies exploring the association of maximum standardized uptake value (SUVmax), metabolic tumor volume (MTV), and total lesion glycolysis (TLG) with overall survival (OS) and progression-free survival (PFS) in STS were included. Pooled hazard ratio (HR) with 95% confidence interval (CI) was calculated using random-effects models.

**Results:**

Nineteen studies with 962 patients were included in our meta-analysis. Among these, 16 studies evaluated the correlation between the SUVmax and OS, 10 studies assessed the relationship between MTV and OS, 9 studies examined the association of TLG with OS, and 8 studies investigated the prognostic value of SUVmax in relation to PFS. The pooled HRs of SUVmax, MTV, and TLG for OS were 1.17 (95% CI: 1.07–1.27),1.87 (95% CI: 1.16–3.03), and 2.00 (95% CI: 0.99–4.01), respectively. For PFS, the pooled HR of SUVmax was1.62 (95% CI: 1.14–2.31).

**Conclusion:**

This meta-analysis indicates that the metabolic parameter SUVmax derived from ^18^F-FDG PET/CT is significantly associated with poor prognosis in STS, for both OS and PFS. Additionally, MTV was significantly correlated with poor OS, whereas TLG did not show a significant relationship with prognosis in patients with STS.

## Introduction

Soft tissue sarcoma (STS) is a heterogeneous group of malignancies originating from mesenchymal tissues, encompassing more than 50 distinct histological subtypes [1]. Despite accounting for merely 1% of adult solid tumors, STS is characterized by aggressive biological behavior and unfavorable clinical outcomes [2]. Accurate prognostication of patients with STS is fundamental for developing individualized treatment strategies. This not only enhances treatment efficacy but also has a substantial impact on improving the quality of life and survival outcomes for those affected by the disease.

^18^F-fluorodeoxyglucose positron emission tomography/computed tomography (^18^F-FDG PET/CT), a cornerstone of molecular imaging, enables non-invasive quantification of tumor glucose metabolism through parameters such as maximum standardized uptake value (SUVmax), total lesion glycolysis (TLG), and metabolic tumor volume (MTV) [3]. In recent years, accumulating evidence has supported its prognostic utility across multiple malignancies, including gastric cancer, lung cancer, esophageal cancer, lymphoma, and so on [4–7]. In the field of sarcoma research, emerging evidence suggests a correlation between ^18^F-FDG PET/CT parameters and survival outcomes. However, significant knowledge gaps remain. Firstly, existing meta-analyses combine data from STS and osteosarcoma, despite the distinct molecular profiles and clinical behaviors of these entities [8, 9]. Secondly, findings from previous studies examining the prognostic value of PET/CT parameters on survival outcomes have been inconsistent. For example, some studies indicate that higher SUVmax is linked to poorer overall survival (OS) [10, 11], while another research suggests that MTV, rather than SUVmax, has a stronger association with both OS and progression-free survival (PFS) [12]. Furthermore, Albano et al. demonstrated that patients with positive restaging PET/CT scans after primary treatment exhibited significantly shorter OS compared to those with negative scans. However, semiquantitative metabolic parameters—including SUVmax, MTV, and TLG—failed to show significant prognostic value for either PFS or OS in their cohort [13]. These discrepancies underscore the urgent need for a rigorous synthesis of the evidence.

Therefore, we aimed to perform a systematic review and meta-analysis to assess the role of ^18^F-FDG PET/CT in prognostic prediction for STS, with the goal of providing a valuable reference for the development of clinical treatment plans.

## Methods

This study was conducted under the guidance of the Preferred Reporting Items for Systematic Reviews and Meta-analyses (PRISMA) guidelines (PROSPERO identifier CRD42024531367) [14]. The literature search, quality control, and data extraction were performed independently by two authors (SLL and RB).

### Search strategy

A systematic search was conducted for all possibly peer-reviewed articles using PubMed, Embase, and the Cochrane Library from database inception until January 1st, 2024. The following keywords were used: ((((((((((((((Sarcomas) OR (Sarcoma, Soft Tissue)) OR (Sarcomas, Soft Tissue)) OR (Soft Tissue Sarcoma)) OR (Soft Tissue Sarcomas)) OR (Sarcoma, Epithelioid)) OR (Epithelioid Sarcoma)) OR (Epithelioid Sarcomas)) OR (Sarcomas, Epithelioid)) OR (Sarcoma, Spindle Cell)) OR (Sarcomas, Spindle Cell)) OR (Spindle Cell Sarcoma)) OR (Spindle Cell Sarcomas)) AND ((((((((((((((((((((“Positron Emission Tomography Computed Tomography“[Mesh]) OR (PET-CT Scan)) OR (PET-CT Scans)) OR (Scan, PET-CT)) OR (Scans, PET-CT)) OR (PET CT Scan)) OR (CT Scan, PET)) OR (CT Scans, PET)) OR (PET CT Scans)) OR (Scan, PET CT)) OR (Scans, PET CT)) OR (CT PET)) OR (Positron Emission Tomography-Computed Tomography)) OR (PET-CT)) OR (CT PET Scan)) OR (CT PET Scans)) OR (PET Scan, CT)) OR (PET Scans, CT)) OR (Scan, CT PET)) OR (Scans, CT PET))) AND ((((((“Prognosis“[Mesh]) OR (Prognoses)) OR (Prognostic Factors)) OR (Prognostic Factor)) OR (Factor, Prognostic)) OR (Factors, Prognostic)).

### Inclusion criteria and exclusion criteria

According to the PICOS (Patient/Intervention/Comparator/Outcome/Study design) framework, the inclusion criteria were as follows: (1) patients with newly diagnosed STS (2), SUVmax, MTV, or TLG measured on pretreatment ^18^F-FDG PET/CT as the intervention (3), no comparator on this study (4), OS or PFS as outcomes, and (5) study design as original articles. Exclusion criteria were: (1) Studies involving other types of tumors without extractable data specific to STS (2), studies that did not provide sufficient data to extract hazard ratios (HRs) for the association between PET/CT parameters and survival outcomes, or (3) patients with other malignant tumors.

### Data extraction and quality evaluation

This meta-analysis extracted the following data from the included studies: first author, and the publication year, country and design of the study, number and age of participants, PET/CT parameters, follow-up duration, and study survival outcomes. OS was defined as the time from diagnosis to death or the date of the last follow-up, while PFS was defined as the time from diagnosis to disease recurrence or progression or the date of the last follow-up. Event-free survival, disease-free survival, and recurrence-free survival reported in the included studies were reclassified as PFS for consistency. HR with 95% confidence intervals (CI) for OS and PFS were also extracted from the studies. If direct data were unavailable, Engauge Digitizer software (version 12.1) was used to extract relevant data from Kaplan-Meier (K-M) survival curves, and the method by Tierney and his colleagues was applied to calculate HR and 95% CI [15].

The Newcastle-Ottawa Scale was used to evaluate the quality of studies, which has eight items with three groups. The total score ranged from 0 to 9, with higher-quality studies receiving higher scores [16]. Any disagreements were discussed by all the authors.

### Statistical analysis

All statistical analyses were performed with R software (R version 4.2.3) and Stata version 14.0 software (Stata Corp, College Station, TX, USA). The HRs with 95% CIs were calculated to assess the prognostic value of PET/CT parameters for STS. The I^2^ was selected to evaluate the heterogeneity among the included studies [17]. When the heterogeneity was more than 50%, a random-effects model was selected, and conversely, a fixed-effect model was used [18]. Subgroup analyses were performed based on country, sample size, and analysis methods. Sensitivity analysis was conducted by omitting one study at a time to detect any significant changes in the obtained results. Funnel plots, as well as Egger’s and Begg’s regression asymmetry test, were employed to evaluate publication bias. All reported probabilities (*P* values) were two-sided, with *P* < 0.05 considered statistically significant.

## Results

### Characteristics of the included studies

**Fig. 1:**
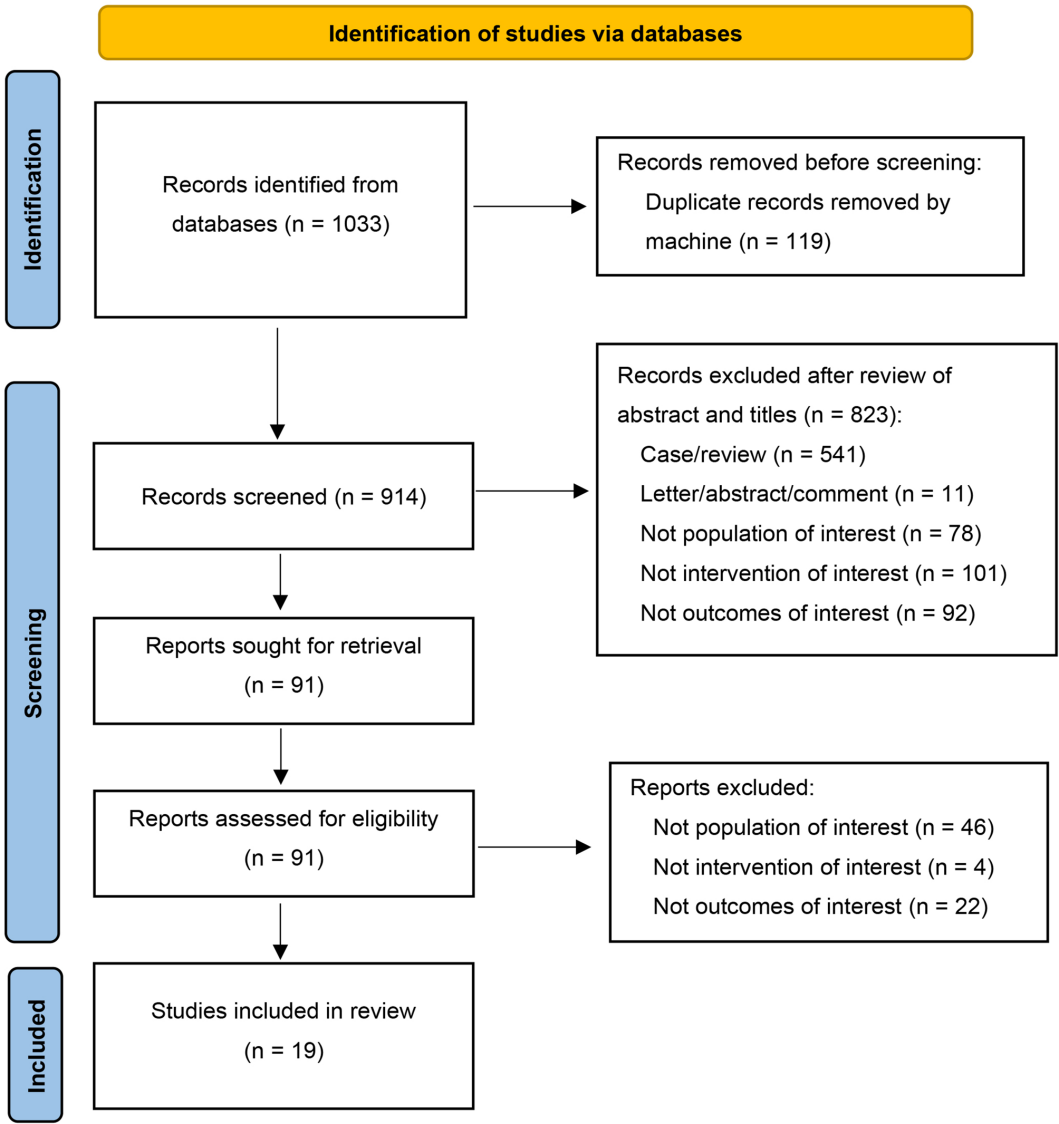
Flow diagram of literature search

There were 1033 studies identified and 19 records were finally included in our analysis (Fig. 1). General detailed characteristics and technical features of eligible studies were shown in Tables 1 and 2. All studies were retrospective, with patients undergoing ^18^F-FDG PET/CT scans prior to the initiation of treatment. A total of ten different countries contributed to the studies, with nearly half originating from Korea (*n* = 8). Significant variations in PET/CT instrumentation were observed across studies. The ¹⁸F-FDG uptake period ranged from 40 to 75 min post-injection, with administered activities typically maintained at approximately 5 MBq/kg in most investigations.

**Table 1:** General characteristics of included studies in the meta-analysis

Author (year)	Country	Patients (*n*)	Gender (male/ female)	Age (years)^#^	PET/CT parameters	Follow-up (months)	Outcomes
Ha et al. (2017) [10]	Korea	36	16/20	50.0	SUVmax, MTV, TLG	60.0	DSS, OS
Lee et al. (2018) [27]	Korea	16	/	51.5	SUVmax, MTV, TLG	21.0	PFS, OS
Annovazzi et al. (2023) [28]	Italy	51	31/20	62.4	SUVmax, MTV, TLG	50.7	DFS, OS
Hong et al. (2014) [29]	Korea	55	34/21	39.0	SUVmax, MTV, TLG	27.0	OS
Chang et al. (2014) [30]	Korea	20	11/9	35.0	SUVmax, MTV, TLG	73.0	OS
Kalisvaart et al. (2021) [31]	Netherlands	31	20/11	58.0	SUVmax, MTV, TLG	32	OS
Kato et al. (2020) [32]	Japan	16	9/7	68.0	SUVmax, MTV, TLG	16.9	OS
Chen et al. (2022) [33]	China	19	9/10	59.0	SUVmax, MTV, TLG	/	OS
Choi et al. (2013) [34]	Korea	66	40/26	52.1	SUVmax, MTV, TLG	23.7	PFS
Fayolle et al. (2022) [12]	France	101	62/39	7.4	SUVmax, MTV	40.0	PFS, OS
Schuetze et al. (2004) [35]	USA	46	21/25	47.0	SUVmax	46.0	RFS, OS
Nishiyama et al. (2012) [11]	Japan	42	28/14	44.0	SUVmax	32.6	EFS, OS
Park et al. (2017) [36]	Korea	19	0/19	51.0	SUVmax	20.0	DFS, OS
Rhu et al. (2019) [37]	Korea	133	73/60	55.9	SUVmax	/	RFS, OS
Subramaniam et al. (2021) [38]	Australia, USA	58	33/25	63.5	SUVmax	34.8	RFS, OS
Umemura et al. (2017) [39]	Japan	18	12/6	76.5	SUVmax	18.0	OS
Hack et al. (2021) [40]	Switzerland	51	30/21	57.0	SUVmax	47.0	OS
Jo et al. (2022) [41]	Korea	129	62/67	56.4	SUVmax	37.8	OS
Andersen et al. (2015) [42]	Denmark	55	26/29	55.2	MTV, TLG	2.2	OS

Abbreviation: SUVmax, maximum standardized uptake value; MTV, metabolic tumor volume; TLG, total lesion glycolysis; DSS, disease specific survival; OS, overall survival; EFS, event-free survival; DFS, disease-free survival; RFS, recurrence-free survival; PFS, progression-free survival

^#^ The mean or median age

**Table 2:** Technical features of included studies in the meta-analysis

Author (year)	Scanner	Uptake time (minutes)	Injected activity	Measurement of MTV
Ha et al. (2017)	Biograph Sensation 16 /TruePoint 40 system (Siemens Medical Systems, Knoxville, TN, USA) or Discovery STE 8/Discovery 690 system (GE Healthcare, Milwaukee, WI, USA)	60	370–550 MBq (total)	SUV2.5
Lee et al. (2018)	Biograph (Siemens Medical Solutions, Malvern, PA) or a Gemini scanner (Philips Medical Systems, Andover, MA)	45–60	5-5.18 MBq/kg (370–555 MBq, total)	SUV2.5
Annovazzi et al. (2023)	Siemens Biograph 16 (Siemens Healthineers)	60 ± 10 (SD)	5 MBq/kg	SUV2.5 and SUV3.0
Hong et al. (2014)	Discovery LS (GE Healthcare, Milwaukee, WI, USA)	45	5.5 MBq/kg	liver activity
Chang et al. (2014)	Biograph 6 (Siemens Medical Solution, Knoxville, TN).	/	7.4 MBq/kg	SUV2.5
Kalisvaart et al. (2021)	Philips Healthcare (Best, The Netherlands)	60	/	SUVpeak 50%
Kato et al. (2020)	Discovery ST Elite, GE Healthcare, Discovery IQ, GE Healthcare, Discovery PET/CT 610, GE Healthcare, Biograph 16 TruePoint, Siemens Healthcare	58 ± 7.1 (SD)	2–5 MBq/kg (123.1–336.7 MBq, total)	SUV2.5
Chen et al. (2022)	Biograph 16 (Siemens, Germany)	40–60	3.75–5.55 MBq/kg	SUV2.5 and SUV41%
Choi et al. (2013)	Gemini (Philips Medical Systems, Milpitas, CA) or Biograph 40 (Siemens Medical Solutions, Knoxville, TN).	60	5.18 MBq/kg	SUV40%
Fayolle et al. (2022)	General Electric Discovery ST, General Electric Discovery ST710, Philips Gemini Allegro Body, Philips Gemini Big Bore, GE discovery, Siemens biograph 16, Siemens Biograph, Siemens mCT20 flow, Siemens biograph 6.0 Truepoint Hirez, Philips Gemini XLI, or General Electric Discovery ST710	60	/^#^	SUV40%
Schuetze et al. (2004)	GE Advance Positron Tomograph (General Electric, Waukesha, WI)	45	7–10 mCi	/
Nishiyama et al. (2012)	Aquiduo PCA-7000B (Toshiba Medical Systems, Tokyo, Japan)	55–60	370 MBq (total)	/
Park et al. (2017)	Accu-Chek^®^ (Roche, Indianapolis, IN, USA)	60	0.14 mCi/kg	/
Rhu et al. (2019)	Discovery STE (GE Healthcare, Waukesha, WI, USA)	60	5.0 MBq/kg	/
Subramaniam et al. (2021)	/	60–75	3.6 MBq/kg	/
Umemura et al. (2017)	/	/	/	/
Hack et al. (2021)	GE Healthcare DSTX	45–60	2–4 MBq/kg	/
Jo et al. (2022)	Discovery STE, GE Healthcare (Waukesha, WI, USA)	60	5.0 MBq/kg	/
Andersen et al. (2015)	GE Discovery LS, Siemens Biograph Sensation 16, Siemens Biograph 40 TruePoint, Siemens Biograph 64 TruePoint, Siemens Biograph mCT-S 64, Siemens Medical Solutions	60	4 MBq/kg	SUV40%

Abbreviation: MTV, metabolic tumor volume; SUVpeak, peak standardized uptake value

# Using EANM paediatric calculators

Sixteen articles examined the association between SUVmax and OS, while ten articles investigated the relationship between MTV and OS. Additionally, nine studies assessed the correlation between TLG and OS, and eight studies explored the relationship between SUVmax and PFS. The analytical methodologies employed across the studies were diverse; the majority utilized multivariate Cox regression analysis, whereas some applied univariate Cox regression analysis.

### Quality evaluation

According to the Newcastle-Ottawa Scale, all the included studies had clear diagnostic criteria for STS. The average score of the studies was 7.1, with all studies scoring 6 or higher. This indicates that the quality of the literature included in this meta-analysis is acceptable. The sections with lower scores were primarily related to the adequacy and completeness of follow-up duration for survival outcomes (as shown in Table 3).

**Table 3:** Results of quality assessment using the Newcastle–Ottawa scale

Study	Selection	Comparability	Outcome			Total score
			Assessment of outcome	Follow-up long enough for outcomes	Adequacy of follow-up of cohorts	
Schuetze et al. (2004)	3	2	1	1	1	8
Nishiyama et al. (2012)	3	1	1	1	0	6
Choi et al. (2013)	3	2	1	1	1	8
Hong et al. (2014)	3	1	1	1	0	6
Chang et al. (2014)	2	1	1	1	1	6
Andersen et al. (2015)	3	2	1	0	0	6
Ha et al. (2017)	4	2	1	1	1	9
Park et al. (2017)	4	1	1	1	0	7
Umemura et al. (2017)	2	2	1	1	1	7
Lee et al. (2018)	3	2	1	1	1	8
Rhu et al. (2019)	3	2	1	0	0	6
Kato et al. (2020)	3	2	1	1	1	8
Kalisvaart et al. (2021)	3	2	1	1	1	8
Hack et al. (2021)	3	2	1	1	1	8
Subramaniam et al. (2021)	3	1	1	1	0	6
Jo et al. (2022)	3	2	1	1	1	8
Chen et al. (2022)	3	2	1	0	0	6
Fayolle et al. (2022)	3	1	1	1	1	7
Annovazzi et al. (2023)	3	2	1	1	0	7

### Meta-analyses and subgroup analyses

#### Results of SUVmax for OS and PFS

**Fig. 2:**
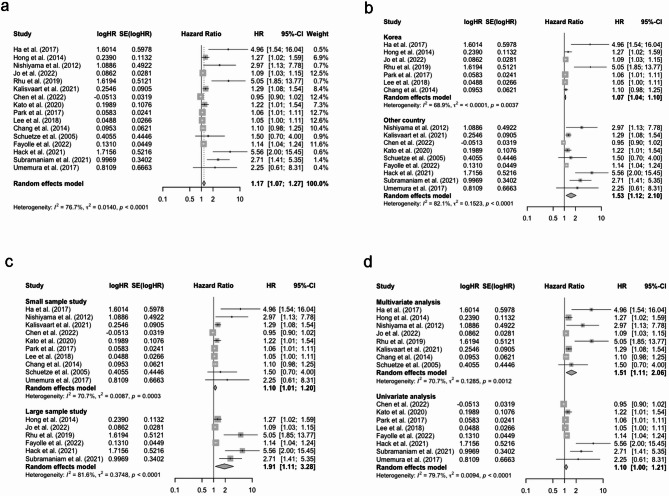
(**a**) Forest plot of pooled HR for OS with SUVmax; (**b**) Subgroup analysis of pooled HR for OS with SUVmax (based on country); (**c**) Subgroup analysis of pooled HR for OS with SUVmax (based on sample size); (**d**) Subgroup analysis of pooled HR for OS with SUVmax (based on analysis method). Abbreviation: HR, hazard ratio; OS, overall survival; SUVmax, maximum standardized uptake value

The prognostic values of SUVmax for OS was assessed in 16 studies. The overall pooled HR of SUVmax was 1.17 (95% CI: 1.07–1.27, Fig. 2a) with significant heterogeneity (I^2^ = 76.7%). Consequently, subgroup analyses were carried out based on country, study sample size, and analysis method to identify the possible sources of heterogeneity. The results showed that heterogeneity decreased slightly in the Korea subgroup (I^2^ = 68.9%), small sample subgroup (I^2^ = 70.7%), and multivariate analysis subgroup (I^2^ = 70.7%), while other subgroups remained to be highly heterogeneous (see Fig. 2b-d).

**Fig. 3:**
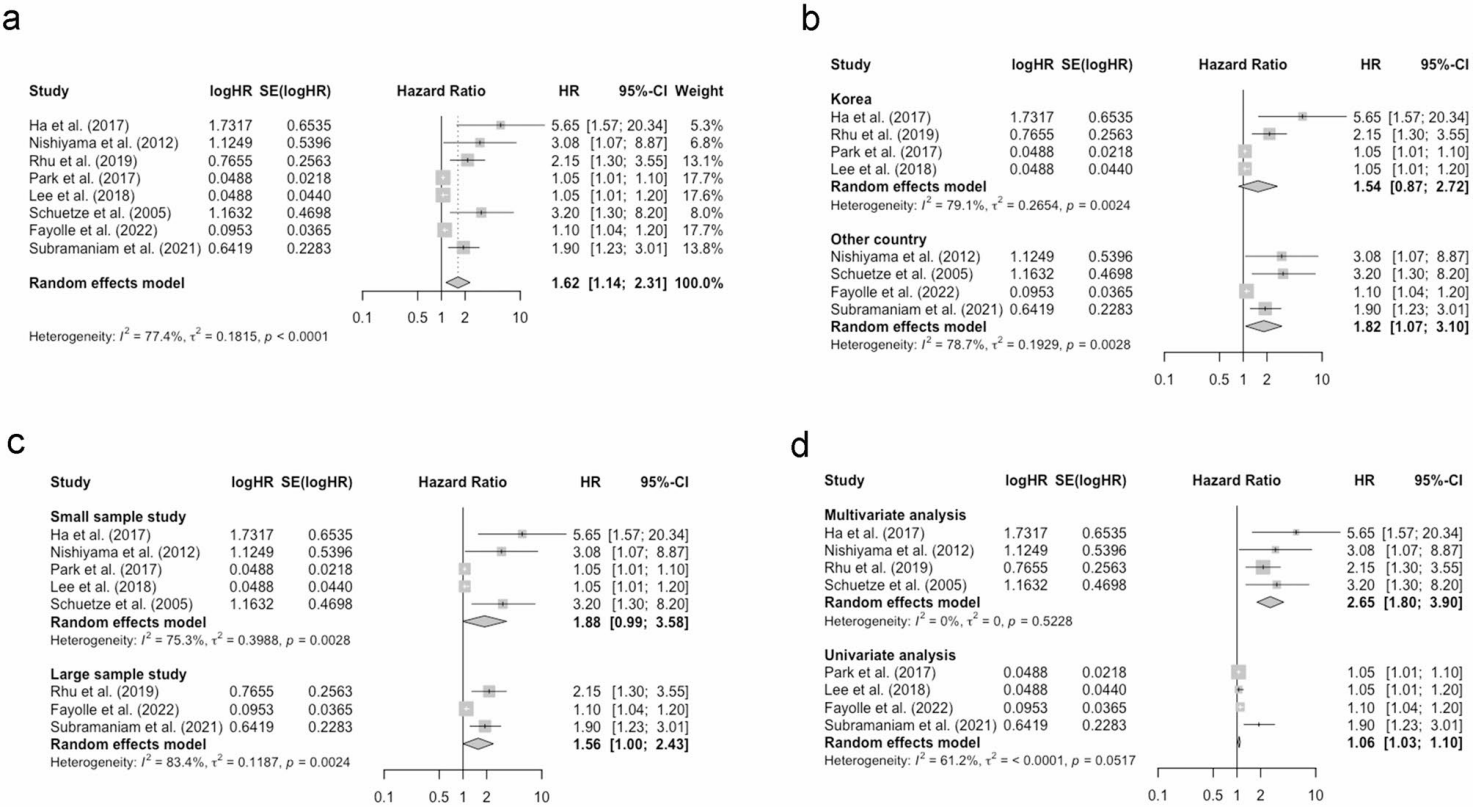
(**a**) Forest plot of pooled HR for PFS with SUVmax; (**b**) Subgroup analysis of pooled HR for PFS with SUVmax (based on country); (**c**) Subgroup analysis of pooled HR for PFS with SUVmax (based on sample size); (**d**) Subgroup analysis of pooled HR for PFS with SUVmax (based on analysis method). Abbreviation: HR, hazard ratio; PFS, progression-free survival; SUVmax, maximum standardized uptake value

A total of eight studies evaluated the prognostic value of SUVmax for PFS, and the pooled HR was 1.62 (95% CI: 1.14–2.31, Fig. 3a) with high heterogeneity (I^2^ = 77.4%). When stratified to analysis method, no significant heterogeneity was present in the multivariate analysis subgroup (I^2^ = 0.0%, Fig. 3d). However, the heterogeneity remained high in different country and sample size subgroups (see Fig. 3b-c).

#### The results of MTV and TLG for OS

**Fig. 4:**
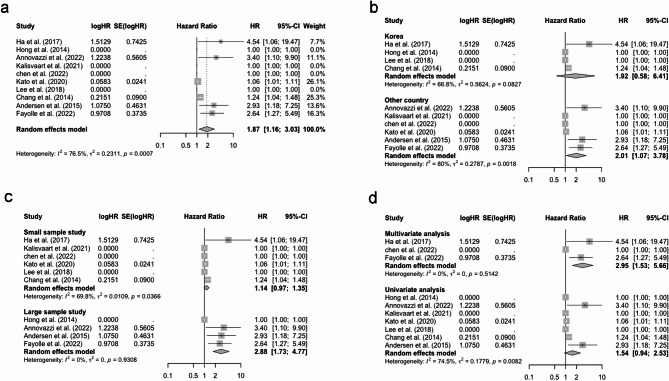
(**a**) Forest plot of pooled HR for OS with MTV; (**b**) Subgroup analysis of pooled HR for OS with MTV (based on country); (**c**) Subgroup analysis of pooled HR for OS with MTV (based on sample size); (**d**) Subgroup analysis of pooled HR for OS with MTV (based on analysis method). Abbreviation: HR, hazard ratio; OS, overall survival; MTV, metabolic tumor volume

The pooled HR of MTV for OS was 1.87 (95% CI: 1.16–3.03), and high heterogeneity was present (I^2^ = 76.5%, Fig. 4a). The results of subgroup analyses revealed t a slight decrease in heterogeneity in the Korea subgroup (I^2^ = 66.8%) while remaining high heterogeneity in the other country subgroup (I^2^ = 80.0%, Fig. 4b). When stratified by study sample size, no significant heterogeneity was found in the large sample subgroup (I^2^ = 0.0%, Fig. 4c). In addition, heterogeneity in the multivariate analysis subgroup was low (I^2^ = 0.0%), while it remained high in the univariate analysis subgroup (I^2^ = 74.5%, Fig. 4d).

**Fig. 5:**
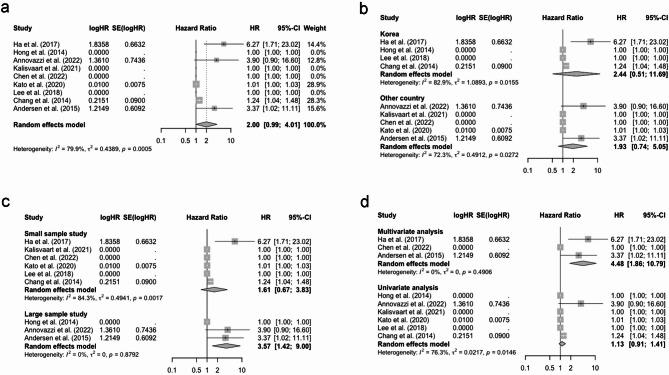
(**a**) Forest plot of pooled HR for OS with TLG; (**b**) Subgroup analysis of pooled HR for OS with TLG (based on country); (**c**) Subgroup analysis of pooled HR for OS with TLG (based on sample size); (**d**) Subgroup analysis of pooled HR for OS with TLG (based on analysis method). Abbreviation: HR, hazard ratio; OS, overall survival; TLG, total lesion glycolysis

For TLG in relation to OS, the pooled HR was 2.00 (95% CI: 0.99–4.01, I^2^ = 79.9%, Fig. 5a). Subgroup analyses indicated decreased heterogeneity in other country (I^2^ = 72.3%), large sample subgroup (I^2^ = 0.0%), and multivariate analysis (I^2^ = 0.0%). However, the heterogeneity remained high in other subgroups (see Fig. 5b-d).

Overall, our meta-analysis demonstrated that high SUVmax were significantly associated with poor OS and PFS in patients with STS. Additionally, MTV was associated with poor OS, while there was no significant correlation between TLG and OS. significant heterogeneity existed in our analysis, and the results of subgroup analyses suggested that the sample size and analysis method may be sources of this heterogeneity.

### Sensitivity analysis and publication bias

**Fig. 6:**
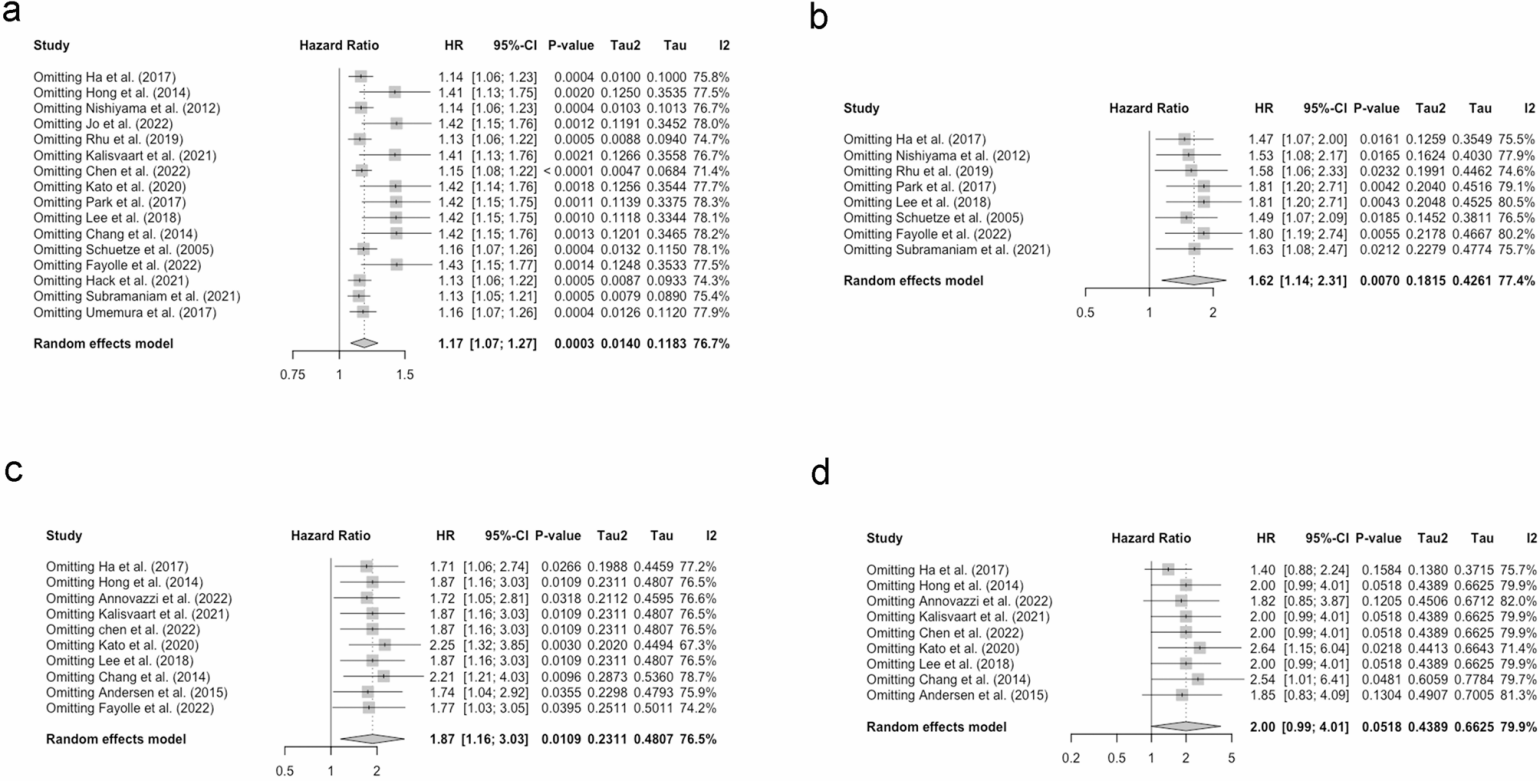
Sensitivity analysis for (**a**) OS with SUVmax, (**b**) OS with MTV, (**c**) OS with TLG, and (**d**) PFS with SUVmax. Abbreviation: HR, hazard ratio; OS, overall survival; SUVmax, maximum standardized uptake value; MTV, metabolic tumor volume; TLG, total lesion glycolysis; PFS, progression-free survival

**Fig. 7:**
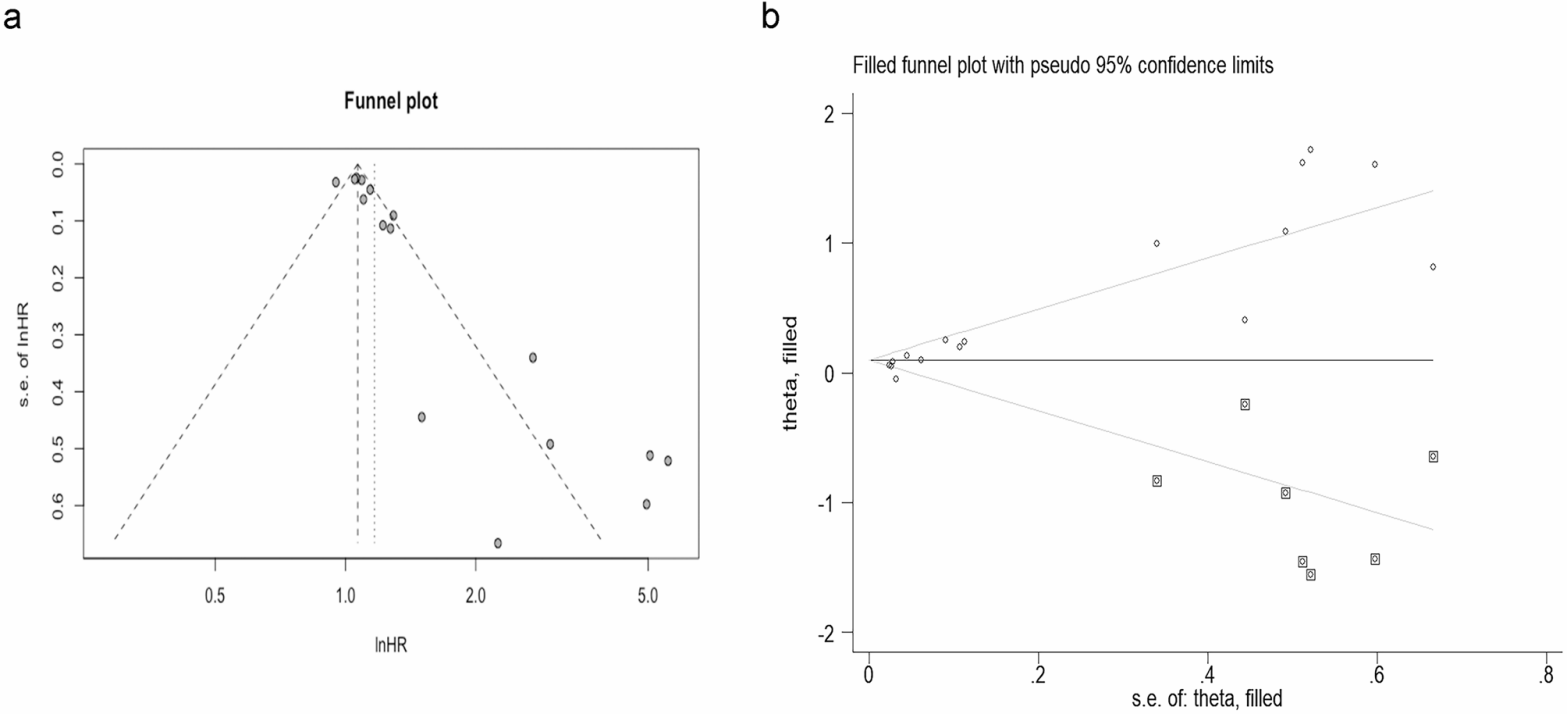
Funnel plots without (**a**) and with (**b**) trimming and filling

Sensitivity analysis indicated that no single study exerted excessive influence on the pooled estimate (see Fig. 6), suggesting that the results of our meta-analysis are relatively reliable. Publication bias was tested using both Egger’s and Begg’s tests, and funnel plots for the correlation of SUVmax with OS were generated. The analysis revealed significant publication bias in the HR of SUVmax for OS (see Fig. 7a, Egger’s *P* = 0.006; Begg’s *P* < 0.001). Consequently, we performed trimming and filling and a symmetrical funnel plot were obtained (Fig. 7b). Notably, no significant change in result was observed after adjusting for publication bias (HR = 1.11, 95% CI: 1.02–1.20), indicating that the overall findings were reliable. Furthermore, no significant publication bias was detected in the HRs of MTV and TLG for OS (both *P >* 0.05 in Egger’s and Begg’s tests).

## Discussion

In this systematic review and meta-analysis, we assessed the prognostic value of SUVmax, MTV, and TLG in patients with STS. The results suggest that a higher SUVmax from ^18^F-FDG PET/CT is associated with poorer OS and PFS in STS. Additionally, a high MTV is significantly linked to short OS, whereas TLG does not demonstrate significant prognostic value for STS.

SUVmax reflects the metabolic activity of tumor lesions and is the most widely utilized PET/CT parameter for disease diagnosis and monitoring treatment response [19]. Our analysis indicates that SUVmax may be a significant prognostic indicator in STS, corroborating findings from a prior meta-analysis [20]. This association likely stems from the biological basis of FDG avidity, as increased glycolytic metabolism in aggressive STS subtypes signifies heightened cellular proliferation and metastatic potential, driven by upregulated glucose transporter 1 expression and enhanced hexokinase II activity [21–23]. As a quantitative volumetric parameter derived from ^18^F-FDG PET/CT, MTV provides critical information about both the spatial extent and glycolytic activity of neoplastic tissue. Elevated MTV values correlate with increased tumor cellularity and amplified glucose avidity (as reflected by SUVmax), suggesting a potential association with more aggressive biological behavior and adverse clinical outcomes in various malignancies [20, 24]. TLG, calculated as the product of MTV and the mean standardized uptake value, integrates both volumetric and metabolic characteristics of tumors, thereby offering a comprehensive assessment of their metabolic burden [25, 26]. However, our study found that TLG did not exhibit prognostic significance. The HR associated with this finding approached the threshold of significance, and substantial heterogeneity was noted. Therefore, caution should be exercised in interpreting these results, and further investigation is warranted to clarify the prognostic value of TLG in STS.

In this meta-analysis, significant heterogeneity was observed. Subsequent subgroup analyses suggested that this heterogeneity may be attributed to factors such as the variations in analytical methodologies and differences in sample sizes. However, other discrepancies among the studies could also contribute to this observed heterogeneity. Firstly, some studies exclusively recruited participants with a single pathological subtype, such as angiosarcoma or leiomyosarcoma, while others included multiple pathological subtypes in their analyses. Given that prognoses can inherently vary across different subtypes [2], this likely contributed to the observed heterogeneity. Additionally, the status of subjects at initial diagnosis, including the presence of metastasis, and the treatment regimens they underwent were also diverse. These baseline differences may further account for the observed heterogeneity. Furthermore, Significant variation exists in the reported cutoff values for SUVmax, MTV, and TLG across studies. This heterogeneity stems from multiple factors, notably differences in patient populations, study endpoints, and statistical methods. Crucially, variations in PET/CT hardware, acquisition protocols, and measurement approaches represent a fundamental source of this heterogeneity. This is particularly relevant for MTV and TLG, whose calculation is highly sensitive to segmentation methodologies. These discrepancies underscore the importance of standardized protocols and caution when applying single cutoffs across diverse settings. These discrepancies could also represent a fundamental source of heterogeneity.

While this study has thoroughly examined the prognostic relationship between ^18^F-FDG PET/CT and STS, it is crucial to acknowledge certain limitations inherent in our analysis. First, there is a significant degree of heterogeneity among the results. Despite employing a random-effects model, conducting subgroup analyses, and confirming the stability of our findings, caution is warranted in the interpretation of these outcomes. Second, there is prognostic variability among different STS subtypes. The studies included in our review exhibited significant differences in the pathological subtypes addressed, and in some cases, the specific distribution of these subtypes was not clearly defined. This lack of clarity restricts our ability to conduct further subgroup analyses based on subtype categorization, which may impact the final results. Third, several of the included studies relied solely on univariate analyses. Furthermore, in some instances, the original HRs were not readily available and required extraction using appropriate methodologies, which could introduce potential confounding factors into the results. Lastly, all articles included in our review were retrospective analyses, with some featuring small sample sizes. Variations in study design and PET/CT analysis may further influence the findings of this meta-analysis.

## Conclusion

In conclusion, this meta-analysis suggests that ^18^F-FDG PET/CT parameters of SUVmax and MTV, but not TLG are significant prognostic factors in patient with STS. Future, well-designed prospective studies with larger sample sizes are warranted to verify the prognostic value of 18F-FDG PET/CT in patients with STS.

## Data Availability

No datasets were generated or analysed during the current study.
